# Comparison of fatigue and fatigability correlates in Korean breast cancer survivors and differences in associations with anxiety, depression, sleep disturbance, and endocrine symptoms: a randomized controlled trial

**DOI:** 10.1186/s12885-021-08575-0

**Published:** 2021-07-26

**Authors:** Min Kyeong Jang, Jeehee Han, Sung Hae Kim, Yun Hee Ko, Soo Yeon Kim, Sue Kim

**Affiliations:** 1 College of Nursing, Mo-Im Kim Nursing Research Institute, Yonsei University, Seoul, Korea, 50-1 Yonsei-ro, Seodaemun-gu, Seoul, 03722 South Korea; 2 Red Cross College of Nursing, Chung-Ang University, Seoul, Korea; 3 Department of Nursing, College of Health, Welfare and Education, Tongmyong University, Busan, South Korea

**Keywords:** Fatigue, Fatigability, Breast Cancer, Exercise, Anxiety, Depression, Sleep disturbance, Endocrine symptoms

## Abstract

**Background:**

Fatigue is one of the most common and burdensome symptoms experienced by cancer patients. In interventions intended to reduce fatigue in such patients, fatigability, or perception of fatigue contextualized to activities of fixed intensity and duration, may also be measured. This study investigated the effects of a 15-month intervention on fatigue and fatigability in breast cancer survivors (BCS); explored the fatigue-fatigability relationship; and evaluated the impacts of fatigue and fatigability on anxiety, depression, sleep disturbance, and endocrine symptoms.

**Methods:**

A randomized controlled trial design was applied to an exercise program called BLESS (Better Life after cancer, Energy, Strength, and Support). The intervention included this 12-week exercise program and four follow-up contacts intended to promote exercise adherence over the following year. Participants were women aged 20 to 69 who had been diagnosed with stage I, II, or III breast cancer; had completed active treatment; and had moderate or higher fatigue. At the completion of the intervention, the survey responses of 40 BCS were evaluated using the chi-square test and multiple regression analysis. The Korean versions of the Revised Piper Fatigue Scale and Pittsburgh Fatigability Scale were used to measure fatigue and fatigability, respectively.

**Results:**

There was no significant difference in fatigue or fatigability between the experimental and control groups at intervention completion. However, the control group showed a stronger association than the experimental group between fatigue and physical fatigability. In the control group, fatigue and fatigability were significantly associated with anxiety, depression, sleep disturbance, and endocrine symptoms. In the experimental group, only the cognitive/mood fatigue score and depression were significantly associated. Only endocrine symptoms influenced mental fatigability (B = − 0.185, *P* < 0.05), and only depression influenced cognitive/mood fatigue (B = 1.469, *P* < 0.05).

**Conclusions:**

Fatigue and fatigability showed different correlations with cancer-related symptoms after the exercise intervention. Future assessments of fatigability in intervention studies will allow measurement of the spectrum of patients’ abilities to overcome fatigue at various physical activity levels while capturing different aspects of cancer-related symptoms.

**Trial registration:**

This study was retrospectively registered on Clinical Research Information Service (KCT0005763; date of registration: 31/12/2020).

## Introduction

Breast cancer is a growing global health burden, as it is the most commonly diagnosed cancer and the leading cause of cancer mortality in women globally [1]. Survival rates for breast cancer are also increasing due to advances in screening and treatment strategies, but with extended survival comes a growing symptom burden. The symptom most frequently reported by breast cancer survivors (BCS) is fatigue, which is further related to anxiety, depression, sleep disturbance, and limitations on survivors’ quality of life [2]. Although fatigue is known to be associated with other cancer-related symptoms as well as survival rates [3, 4], it is challenging to precisely understand and assess fatigue, first because it has a considerable range of definitions and multidimensional characteristics, and second because fatigue changes over time during survivorship. One way to precisely measure fatigue is in terms of level of physical activity, and the term “fatigability” has been used to refer to the fatigue perceived by an individual at certain activity levels [5, 6].

Considering the fact that cancer patients’ health conditions can change at any time and the importance of sensitively measuring their cancer-related fatigue (CRF), measurements of fatigability can play a key role in understanding changes in patients’ perceived fatigue while accounting for self-pacing of activity. In oncology, the concept of fatigability has recently emerged as a way to accurately measure an individual’s potential ability to overcome fatigue, both physical and mental [7–10]. The usefulness of this concept for understanding CRF has been illustrated by previous research findings. For example, in a recent longitudinal study, after adjusting for age, sex, comorbidities, and body mass index, a history of cancer was associated with greater fatigability [8]. Another recent study that focused on breast cancer found that over 71% of BCS had physical fatigability and that 53% experienced mental fatigability; furthermore, physical fatigability was a significant predictor of survivors’ quality of life [9]. Although fatigability is increasingly accepted as an important predictor or early marker of perceived fatigue levels that encompasses physical activity type, duration, and intensity, no known studies have examined fatigability or its relationship with cancer-related symptoms in Korean BCS. Consequently, assessment of fatigability in BCS and its impact on their cancer-related symptoms is called for.

Exercise interventions have been proven to alleviate fatigue while improving physical outcomes during and after breast cancer treatment. In a previous umbrella review of 24 systematic reviews on breast cancer and exercise, 21 studies (87.5%) reported that exercise reduced CRF and that both resistance and aerobic exercise helped to relieve fatigue symptoms in BCS [11]. Moreover, in a systematic review of randomized controlled trials (RCTs) including 36 studies, combinations of aerobic and resistance exercise showed significant associations with improved quality of life in BCS [12]. In addition, exercise has been associated with reduced anxiety, depression, sleep disturbance, and endocrine symptoms in cancer survivors [13, 14].

Although many researchers have studied the benefits of exercise for controlling fatigue and other cancer-related symptoms, few studies have examined the effects of long-term adherence to an exercise program on both fatigue and fatigability. In one previous study [15], no significant differences in fatigue were found between intervention and control groups at baseline; anxiety, depression, sleep quality, physical activity, and quality of life also showed no significant differences between the groups. In a second study [16], fatigue total and subdomain scores at baseline and at three post-exercise program timepoints displayed no significant differences in analyses for group effects and group-time effects, but the data were not used to examine fatigability or the relationship between fatigue and fatigability. While fatigability is an emerging concept in oncology research and various exercise interventions have been developed for breast cancer patients, to our knowledge, no study has explored the relationships between fatigue and fatigability in BCS after a long-term exercise intervention. Furthermore, the relationship of fatigability to cancer-related symptoms such as anxiety, depression, sleep disturbance, and endocrine symptoms remains uncertain.

To help fill the current research gaps in this area, our primary aims were (1) to identify the effects of an intervention combining an exercise program with adherence follow-ups on BCS’ fatigue and fatigability; (2) to explore the relationship between fatigue and fatigability; and (3) to compare the impacts of both fatigue and fatigability on anxiety, depression, sleep disturbance, and endocrine symptoms between the experimental and control groups.

## Methods

### Study design

This follow-up study employed a two-armed, prospective, RCT design. This study was performed to identify the effects of an exercise adherence intervention called BLESS (Better Life after cancer, Energy, Strength, and Support) [17] at one-year follow-up and assess the effects of long-term exercise on survivors’ fatigue and fatigability, as well as factors related to anxiety, depression, sleep disturbance, and endocrine symptoms in the participants.

### Study participants and setting

Study participants were recruited using convenience sampling and snowball sampling from Yonsei University Health Systems Severance Hospital, Seoul, South Korea. Under the study inclusion criteria, participants had (1) to be women aged 20 to 69 years, (2) to have been diagnosed with stage I, II, or III breast cancer between 6 months and 5 years before study initiation, (3) to have completed surgery and other treatments such as chemotherapy and radiation, and (4) to show moderate or higher fatigue (i.e., a score ≥ 4 on a rating scale of 0 to 10 during participant recruitment). Eligible participants were randomly assigned to the experimental group or control group applying computer-generated random number sequencing (1:1 ratio). The participants and the research team were not blinded with respect to group assignments. Based on an effect size of .80 in the previous similar study [18], the sample size was estimated as 46 participants. The BLESS researchers randomly assigned 50 BCS to an experimental group (*n* = 24) and a control group (*n* = 26) at baseline. Because of cancer recurrence, death, family illness, and loss during follow up, data for 40 participants who completed a follow-up survey after 1 year of adherence to the exercise program were analyzed in this study.

### Study procedure

This study was registered on Clinical Research Information Service (KCT0005763; date of registration: 31/12/2020) and approved by the institutional review board at the Yonsei University Health Systems Severance Hospital (#4–2017-0164). In the 12-week active component of the BLESS intervention, both aerobic and resistance exercises were conducted by two physical trainers and an oncology practitioner. Of the total 12 weeks, the first 6 weeks involved in-person supervised exercise and the last 6 weeks consisted of home-based exercise. During the first 6 weeks, the experimental group attended eight supervised exercise sessions, which combined both resistance and aerobic exercise. Participants also took part in weekly small-group meetings to strengthen bonding and bridging social capital among survivors. Exercise intensity was gradually increased from light to vigorous, and each supervised exercise session lasted approximately 60 min. To encourage adherence, exercise video clips were provided in the experimental group. After the experimental group had completed the 12-week active exercise program, the control group was offered the program. During the 12-week program, text messages regarding fatigue, exercise-related information, and health-related motivations were provided to both the experimental and control groups every week. Also, exercise encouragement messages were provided only to the experimental group before and after the 12-week BLESS program.

After completion of the 12-week active exercise program, we followed up with both groups immediately after the program and at 1, 6, and 12 months after the program. Also, after the end of the program, we made an effort to minimize attrition and maximize exercise adherence for both groups by providing special activities (e.g., candle making, calligraphy sessions, and other activities) at the four follow-up measurement points. The BLESS protocol has been thoroughly described by Kim et al. (2019) [17].

### Instruments

#### Fatigue

To measure fatigue, the Korean version of the Revised Piper Fatigue Scale (K-R-PFS) was used. This K-R-PFS instrument has a total of 19 items with four subscales: behavioral/severity (6 items), affective (4 items), sensory (4 items), and cognitive/mood (5 items). Each item measures the degree of fatigue from 0 to 10. The sum of the item scores is divided by the total number of items to calculate the total score in the 0–10 range. Higher total scores indicate higher fatigue. In a previous study, the reliability of the total fatigue score for Korean BCS was shown by a Cronbach’s alpha of 0.93 [19], and this study also had high reliability (Cronbach’s alpha = 0.96).

#### Fatigability

Fatigability was measured using the Korean version of the Pittsburgh Fatigability Scale (K-PFS) [9]. This tool measures the degree of self-perceived fatigue immediately after high, moderate, or sedentary activities, defined according to the activity level (metabolic equivalents). Ten items are used to measure physical fatigability and mental fatigability, respectively. A score of 0 points means “no fatigue” and 5 points means “greater fatigue.” A higher score indicates that a respondent more readily feels fatigue. In earlier research using the PFS, a physical fatigability score ≥ 15 indicated high physical fatigability, and a mental fatigability score ≥ 13 indicated high mental fatigability [20–22]. Also, in a previous study [9], the PFS’s reliability for physical and mental fatigability with Korean BCS was confirmed by Cronbach’s alpha values of 0.87 and 0.86, respectively. In this study, the Cronbach’s alpha values for physical and mental fatigability were 0.81 and 0.81, respectively.

#### Anxiety and depression

Anxiety and depression were measured using the Korean version of the Hospital Anxiety and Depression Scale (K-HADS). The K-HADS consists of seven items for anxiety and seven items for depression, rated from 0 points (“not at all”) to 3 points (“very often”). Higher scores indicate higher levels of anxiety and depression. Within the scale of 0–21 points for each condition, a normal status is interpreted as 0–7 points, 8–10 points as suspected anxiety/depression, and more than 11 points as anxiety/depression. The reliability of the tool in a previous Korean study was confirmed by Cronbach’s alpha values of 0.89 for anxiety and 0.86 for depression [23]. The Cronbach’s alpha values in this study were 0.88 for anxiety and 0.75 for depression, respectively.

#### Sleep disturbance

Sleep disturbance and quality were measured using the Korean version of the Pittsburgh Sleep Quality Index (K-PSQI). The K-PSQI consists of a total of 19 items with seven subdomains: sleep latency, subjective sleep quality, sleep duration, habitual sleep efficiency, sleep disturbance, use of sleeping medication, and daytime dysfunction. In each domain, a higher score corresponds to a lower quality of sleep. A sleep problem for the general population is indicated by a score of more than 5, while a sleep problem for breast cancer patients is indicated by a score of more than 8. This study used the score of 8 to define sleep problems in BCS. The reliability of this tool in a previous Korean study was shown by a Cronbach’s alpha of 0.86 [24], while Cronbach’s alpha was 0.70 in this study.

#### Endocrine symptoms

Endocrine symptoms, reflecting menopausal and sexual symptoms, were measured using the Korean version of the Functional Assessment of Cancer Therapy–Endocrine Symptoms (FACT-ES) instrument. The tool consists of 46 items in five subdomains: physical well-being (7 items), social/family well-being (7 items), emotional well-being (6 items), functional well-being (7 items), and endocrine symptom scale (19 items) [25]. The instrument uses a 5-point Likert scale ranging from 0 to 4; 0 means “not at all” and 4 means “very much.” In the present study, its reliability was shown by Cronbach’s alpha values ranging from 0.80 to 0.93.

### Statistical analyses

Data were analyzed using STATA IC version 16. Descriptive statistics as well as the independent t-test, the chi-square test, and Pearson’s correlation coefficients were employed to identify general participant characteristics and the relationships between fatigue and fatigability. The factors that affected the relationships of fatigue and fatigability with anxiety, depression, sleep disturbance, and endocrine symptoms were analyzed with multiple regression. To guide selection of variables to be included in multivariable analyses, the homogeneity test of covariates was first applied, and no significant difference between groups was found. Then, based on previous studies that reported relationships among cancer-related symptoms, our research team chose the variables for the multiple linear regression model to confirm those studies’ findings. All tests were two-tailed, with statistical significance set at an alpha level of 0.05.

## Results

### General characteristics

Table 1 summarizes the demographic and clinical characteristics of the study participants in both the experimental group (*n* = 21) and control group (*n* = 19). The average age of participants was similar for the experimental group (50 years) and the control group (48 years). Most participants in both groups had stage II breast cancer (experimental group: 57.1% vs. control group: 68.4%). The entire experimental group and all but one participant in the control group received chemotherapy, and all but four participants in both groups completed radiation therapy. No statistically significant differences were observed between the two groups with regard to demographic or clinical characteristics. Also, clinical and demographic characteristics showed no significant relationships with fatigue or fatigability.

**Table 1:** Demographics and clinical characteristics

Characteristic	Exp. (*n* = 21)	Cont. (*n* = 19)	χ^2^/t (p)
	Mean ± SD (range) N (%)	Mean ± SD (range) N (%)	
Age (years)	49.86 ± 7.9 (34–67)	47.63 ± 6.96 (33–58)	.937(.355)
30–39	1 (4.8)	2 (10.5)	2.573 (.462)
40–49	10 (47.6)	11 (57.9)	
50–59	8 (38.1)	6 (31.6)	
60–69	2 (9.5)	0 (0)	
Marital status			
Married	15 (71.4)	12 (63.2)	.311(.577)
Unmarried	6 (28.6)	7 (36.8)	
Monthly income, 10,000 KRW			
< 300 ($2,660)	11 (52.4)	9 (47.4)	.100 (.752)
≥ 300 ($2,660)	10 (47.6)	10 (52.6)	
(USD dollars)			
Employment status			
No	15 (71.4)	10 (52.6)	1.503(.220)
Yes	6 (28.6)	9 (47.4)	
Education level			
< Middle school	1 (4.8)	1 (5.3)	3.713(.156)
High school	14 (66.6)	7 (36.8)	
≥ College	6 (28.6)	11 (57.9)	
Children			
No	5 (23.8)	4 (21.1)	.043(.835)
Yes	16 (76.2)	15 (78.9)	
Stage			
I	5 (23.8)	5 (26.3)	1.744(.418)
II	12 (57.1)	13 (68.4)	
III	4 (19)	1 (5.3)	
Surgery type			
Mastectomy	4 (19)	5 (26.3)	.302 (.583)
Lumpectomy	17 (81)	14 (73.7)	
Time since diagnosis			
< 1 year	8 (38.1)	8 (42.1)	.575(.750)
1–2 years	11 (52.4)	8 (42.1)	
≥ 2 years	2 (9.5)	3 (15.8)	
Chemotherapy			
None	0 (0)	1 (5.3)	1.134(.287)
Past	21 (100)	18 (94.7)	
Radiation therapy			
None	1 (4.8)	3 (15.8)	1.206(.235)
Past	20 (95.2)	16 (84.2)	
Endocrine therapy			
No	9 (42.9)	8 (42.1)	.002(.962)
Yes	12 (57.1)	11 (57.9)	
Targeted therapy			
No	10 (47.6)	15 (78.9)	4.483(.106)
Yes	10 (47.6)	4 (21.1)	
Unknown	1 (4.8)	0 (0.0)	

*Abbreviations*: *Cont* Control group, *Exp* Experimental group, *KRW* Korean won (1160 KRW = approximately 1 US)

### Descriptive analyses of fatigue and fatigability

Table 2 summarizes the comparison of fatigue scores between the experimental and control groups at baseline and post-intervention. Following the intervention, the mean total fatigue scores measured using the K-R-PFS were 4.52 (SD: 1.93) for the experimental group and 4.23 (SD: 2.18) for the control group. Both before and after the intervention, the total fatigue scores and all subscale scores showed no significant differences between the experimental and control groups.

**Table 2:** Comparison of fatigue score between experimental and control groups at baseline and following intervention

Fatigue	Fatigue subscales	Experimental group	Control group	t (p)
		Mean ± SD (range)	Mean ± SD (range)	
Pre-intervention fatigue	Total fatigue score	5.23 ± 1.57	5.61 ± 1.84	−0.707(.484)
	Behavioral/severity fatigue	5.52 ± 1.45	5.55 ± 2.36	−0.06 (.953)
	Affective fatigue	5.83 ± 2.14	6.25 ± 2.67	−0.546(.588)
	Sensory fatigue	4.81 ± 2.41	5.97 ± 1.67	−1.758(.087)
	Cognitive/mood fatigue	4.73 ± 2.00	4.87 ± 1.84	−0.23 (.819)
Post-intervention fatigue	Total fatigue score	4.52 ± 1.93	4.23 ± 2.18	0.441(.662)
	Behavioral/severity fatigue	4.46 ± 2.27	4.02 ± 2.83	0.541(.592)
	Affective fatigue	5.85 ± 2.56	5.51 ± 2.52	0.412(.683)
	Sensory fatigue	4.06 ± 2.48	4.28 ± 2.20	−0.291(.773)
	Cognitive/mood fatigue	3.91 ± 1.53	3.75 ± 2.36	0.268(.790)

Following the intervention, the mean physical fatigability scores measured using the K-PFS were 24.42 (SD: 5.68) for the experimental group and 22.89 (SD: 9.68) for the control group, and the mean mental fatigability scores were 21.47 (SD: 7.75) for the experimental group and 18.67 (SD: 9.27) for the control group. The experimental and control groups’ fatigability scores did not differ significantly.

Table 3 shows the correlations between fatigue and fatigability by group. Interestingly, the experimental and control groups exhibited different correlations. The experimental group showed no correlation between total fatigue and physical or mental fatigability; however, the control group showed a high positive correlation between total fatigue and physical fatigability (*r* = 0.663, *P* < 0.01). With respect to the fatigue subscales, only sensory fatigue in the experimental group had a positive correlation with physical fatigability (*r* = 0.481, *P* < 0.05) or mental fatigability (*r* = 0.460, *P* < 0.05). In the control group, behavioral (*r* = 0.611, *P* < 0.01), affective (*r* = 0.543, *P* < 0.05), and cognitive/mood (*r* = 0.513, *P* < 0.05) fatigue had moderate positive correlations with physical fatigability.

**Table 3:** Correlations between fatigue and fatigability

Fatigability	Fatigue	Experimental group	Control group
Physical fatigability	Total fatigue score	.408	.663**
	Behavioral/severity fatigue	.445	.611**
	Affective fatigue	.261	.543*
	Sensory fatigue	.481*	.513*
	Cognitive/mood fatigue	.164	.527*
Mental fatigability	Total fatigue score	.414	.425
	Behavioral/severity fatigue	.433	.333
	Affective fatigue	.231	.405
	Sensory fatigue	.460*	.352
	Cognitive/mood fatigue	.274	.436

*p < 0.05, **p < 0.01

**Fig. 1:**
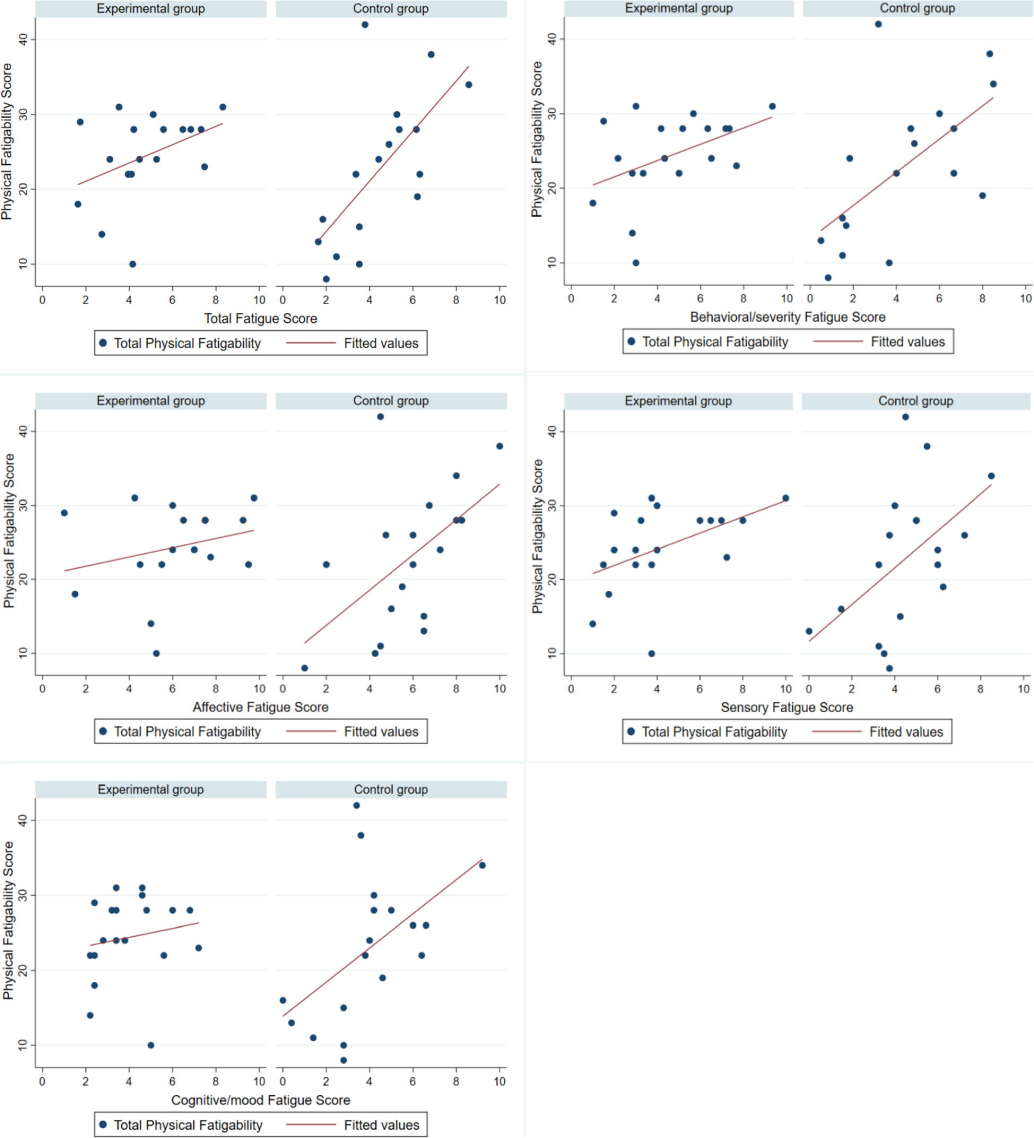
Differences in Fatigue and Physical Fatigability Associations and Distribution of Scores Between Groups

A scatter plot is presented to illustrate differences in fatigue and physical fatigability associations and the distribution of scores between the groups (Fig. 1). With respect to the association between total fatigue and physical and mental fatigability, the control group showed a stronger association than the experimental group between total fatigue and physical fatigability. The control group also showed stronger associations between the four fatigue subdomains and physical fatigability. In addition, the control group distribution remained quite close to a diagonal line of best fit, but the experimental group distribution did not show a linear relationship.

### Descriptive analyses of anxiety, depression, sleep disturbance, and endocrine symptoms

After the intervention, the mean score for anxiety was 7.43 (SD: 3.75) for the experimental group and 6.74 (SD: 3.83) for the control group. Regarding depression, the mean score was borderline (7.71; SD: 3.89) in the experimental group and in the normal range (6.05; SD: 3.05) in the control group. The mean score for sleep disturbance was 8.95 (SD: 2.91) for the experimental group and 7.94 (SD: 3.99) for the control group. Lastly, the mean score for endocrine symptoms was 117.12 (SD: 29.74) for the experimental group and 126.24 (SD: 31.79) for the control group. The differences in symptoms between the experimental and control groups were not statistically significant.

### Relationships among fatigue- and fatigability-related symptoms

#### Fatigue-related symptoms

Table 4 shows the correlations among fatigue- and fatigability-related symptoms by group. In the control group only, fatigue was significantly associated with anxiety, depression, and endocrine symptoms. In that group, anxiety, depression, and endocrine symptoms showed moderate to high correlations with behavioral fatigue (*r* = 0.607, *P* < 0.01; *r =* 0.628, *P* < 0.01; *r =* − 0.782, *P* < 0.001) and total fatigue.

**Table 4:** Differences in the Associations of Fatigue/Fatigability with Anxiety, Depression, Sleep Disturbance, and Endocrine Symptoms by Group

Subdomains	Fatigue										Fatigability			
	Total		Behavioral/severity		Affective		Sensory		Cognitive/mood		Physical		Mental	
Group	Exp	Cont	Exp	Cont	Exp	Cont	Exp	Cont	Exp	Cont	Exp	Cont	Exp	Cont
Anxiety	.390	.549*	.368	.607**	.317	.513*	.321	.310	.372	.366	.046	.464	.244	.546*
Depression	.274	.704**	.187	.628**	.244	.421	.156	.671**	.451*	.663**	−.204	.685**	−.046	.566*
Sleep disturbance	.348	.307	.316	.267	.362	.199	.196	.460	.367	.346	.244	.287	.307	.520*
Endocrine symptoms	−.349	−.767***	−.354	−.782***	−.324	−.657**	−.288	−.635**	−.232	−.555*	−.213	−.709**	−.353	−.505*

*Abbreviations*: *Cont* Control group, *Exp* Experimental group

*p < 0.05, **p < 0.01, ***p < 0.001

(*r =* 0.549, *P* < 0.05; *r =* 0.704, *P* < 0.01; *r =* − 0.767, *P* < 0.001). In the control group, anxiety and endocrine symptoms also showed moderate correlations with affective fatigue (*r =* 0.513, *P* < 0.05; *r =*.

-0.657, *P* < 0.01), and depression and endocrine symptoms showed moderate correlations with sensory fatigue (*r =* 0.671, *P* < 0.01; *r =* − 0.635, *P* < 0.01); in addition, cognitive/mood fatigue was significantly associated with depression (*r =* 0.663, *P* < 0.01) and endocrine symptoms (*r =* − 0.555, *P* < 0.05). However, in the experimental group, the only significant correlation between fatigue and symptoms was between cognitive/mood fatigue and depression (*r =* 0.451, *P* < 0.05).

#### Fatigability-related symptoms

With respect to physical and mental fatigability, depression (*r =* 0.685, *P* < 0.01; *r =* 0.566, *P* < 0.05) and endocrine symptoms (*r =* − 0.709, *P* < 0.01; *r =* − 0.505, *P* < 0.05) showed moderate to high correlations only in the control group. In the control group, anxiety showed a moderate correlation with total mental fatigability (*r =* 0.546, *P* < 0.05). In the experimental group, however, no significant relationships were found between fatigability and symptoms.

### Multiple regression analysis of fatigue and fatigability

The results of the multivariate regression analysis performed to determine the main factors associated with fatigue and fatigability are presented in Table 5. In the multiple regression analysis of total fatigue and the four fatigue subscales, only the model for cognitive/mood fatigue was statistically significant (*F =* 2.94, *P* < 0.05; *R*^2^ = 0.282). Depression was the only factor that influenced cognitive/mood fatigue (B = 1.469, *P* < 0.05). With respect to mental fatigability, the model was statistically significant (*F =* 4.91, *P* < 0.01; *R*^2^ = 0.403). Endocrine symptoms were the only factor that influenced mental fatigability (B = − 0.185, *P* < 0.05).

**Table 5:** Multiple Regression Analysis of Fatigue and Fatigability

Symptoms	Cognitive/mood fatigue			Mental fatigability		
	B	SE	*p*	B	SE	*p*
Anxiety	−.480	.656	0.462	.363	.531	0.500
Depression	1.469	.683	<0.05	−1.103	.546	0.053
Sleep disturbance	.172	.548	0.755	.586	.435	0.188
Endocrine symptoms	−.026	.088	0.769	−.185	.072	<0.05
	*R*^2^ = 0.282, *F =* 2.94, *P* < 0.05			*R*^2^ = 0.403, *F* = 4.91, *P* < 0.01		

## Discussion

Previous studies have shown that exercise intervention is a crucial strategy for reducing CRF, but demonstrating the effectiveness of longer-term adherence to exercise for controlling fatigue has been a challenge. Our previous study found that the experimental and control groups showed no differences in fatigue symptoms immediately after the 12-week active component of the BLESS intervention [15, 16]. With respect to the relationship between exercise and fatigue, in a previous meta-analysis involving 25 RCT studies, exercise interventions showed diminished effectiveness for fatigue symptoms at the 6-month follow-up compared to during and immediately after the interventions [26]. Consistent with those findings, the fatigue levels measured 1 year after the BLESS exercise program did not meaningfully differ from the baseline levels [15, 16]. Given these results, the diminishing impacts of long-term adherence to an exercise program on fatigue, as reported in the literature, may be difficult to explain. However, the current study yielded an interesting finding: the fatigue-fatigability relationship differed between the experimental and control groups. Consequently, fatigability may be a useful measure for identifying the effects of long-term adherence to an exercise program. Furthermore, the factors influencing fatigue and fatigability differed markedly. The finding that fatigue and fatigability were affected by different factors is an important clinical consideration, especially for the development of exercise interventions and the interpretation of cancer-related symptoms.

As shown in Fig. 1, the control group showed a stronger association between fatigue and fatigability than the experimental group. In other words, the control group participants who reported higher fatigue more readily experienced fatigability according to a certain physical activity level, and fatigue was also significantly associated with all cancer-related symptoms in this group. However, in the experimental group, fatigue did not predict fatigability, and fatigue was also not significantly associated with most cancer-related symptoms. This was the case even though fatigue levels did not significantly different between the experimental and control group participants. Since our findings reveal that fatigue and fatigability have different characteristics, the latter can also be useful for evaluating the long-term effects of an exercise intervention on fatigue. According to Eldadah et al. [6], fatigability is a potentially significant parameter, especially in intervention studies. In fact, without considering changes in physical activity levels when assessing perceived fatigue, researchers cannot verify that their interventions are effective for controlling fatigue. Accordingly, assessment of fatigability can provide a more objective understanding of patients’ actual ability to overcome fatigue. On the whole, because the assessment of fatigability can bring beneficial insights to exercise studies involving cancer patients, future intervention researchers should consider including fatigability assessments as an additional dimension of understanding CRF.

Fatigue levels did not significantly differ between the experimental and control groups or between the study baseline and one-year follow-up of the BLESS program. At the one-year follow-up, in the control group only, fatigue and fatigability were found to be significantly associated with anxiety, depression, sleep disturbance, and endocrine symptoms. Interestingly, in the multiple regression analysis, depression was the only factor that influenced cognitive/mood fatigue, while endocrine symptoms were the only factor that influenced fatigability. These findings highlight both the different relationships among fatigue- and fatigability-related symptoms and the differing predictors for fatigue and fatigability. The relationships of fatigue with cancer-related symptoms are well known. For instance, a systematic review of 57 studies revealed evidence that fatigue was positively related to anxiety, depression, and sleep disturbance and negatively related to quality of life [27]. Furthermore, similar to our findings, depressive symptoms were found to be an independent risk factor for fatigue in BCS in a previous study [28]. Given these findings, it seems clear that a relationship exists between fatigue and depression in BCS.

To our knowledge, no previous study has revealed a relationship between fatigability and endocrine symptoms in cancer survivors, but we identified such a relationship. Furthermore, we found that unlike depression, which influenced cognitive/mood fatigue, endocrine symptoms were a predictor of mental fatigability. One possible explanation is that endocrine symptoms were measured using the FACT-ES, which includes quality of life-related domains such as physical, functional, emotional, and social/family well-being, and thus endocrine symptoms related to quality of life may also be related to each other. Similarly, a previous study found that physical fatigability, depressive symptoms, and cognitive impairment impacted quality of life in BCS [9]. Moreover, endocrine symptoms, which include menopausal and sexual symptoms, were reported to influence the relationship between fatigue and quality of life in a previous study involving breast cancer patients receiving adjuvant chemotherapy [29]. Therefore, we believe that these complex correlations observed in our and previous studies may support our finding of a significant relationship between mental fatigability and endocrine symptoms.

Notably, recent fatigability research has provided new epidemiological insights related to fatigability. For instance, in one study, physical fatigability was related to body mass index and interleukin-6 as an inflammatory marker, and women showed significantly higher physical fatigability than men [20]. Furthermore, in a pilot study involving 29 older adults who received 7-T magnetic resonance imaging exams, higher physical fatigability was related to lower brain volume in specific regions such as the hippocampus, putamen, and thalamus [21]. Given the recent findings regarding factors related to fatigability, multiple additional clinical factors need to be explored to determine their relationship to fatigability in cancer survivors.

Our findings reveal meaningful differences in the characteristics of fatigue and fatigability and offer directions for future intervention research, but the study’s limitations should also be noted. Although we measured fatigue and other symptoms at the study baseline and 1 year after the exercise program was completed, fatigability was assessed only once at the completion of the intervention, limiting our ability to compare changes in fatigability. Further studies are needed to explore patterns of fatigability in cancer survivors as well as to compare survivors’ fatigue and fatigability characteristics before, during, and after exercise interventions. Also, no blinding was performed during group allocation of participants, and thus the researchers’ and participants’ attitudes and actions may have been affected by bias. Furthermore, the sample size after 1 year of adherence to the exercise program was small. Because the small sample size limits the generalizability of our results and reduces their statistical power, additional, multicenter studies with large enough samples are needed to obtain more generalizable fatigue and fatigability findings. Finally, although various fatigability measures have been developed, no gold standard exists to assess fatigability in the oncology field. Research and development of fatigability measures for cancer survivors should continue until a gold standard is achieved.

## Conclusion

This study’s findings have important clinical implications for fatigability assessment, delineation of the different characteristics of fatigue and fatigability, and identification of the different factors that influence them. Based on our observation of a significant relationship between mental fatigability and endocrine symptoms, further studies are warranted to explore the impacts of this relationship on BCS. In addition, prospective longitudinal studies are needed to identify changes in fatigability in this population over time. Also, our results provide directions for future exercise intervention studies and highlight the need for fatigability measures to capture the full spectrum of cancer survivors’ physical activity. Although encouraging cancer survivors with fatigue to undertake an exercise intervention did not seem to reduce their fatigue in the long term, their ability to overcome fatigue depending on physical activity level may have changed. Thus, oncology researchers need to be attentive to the differences between fatigue and fatigability, especially in development of exercise interventions and interpretation of fatigue-related symptoms.

## Data Availability

The datasets generated and/or analyzed during the current study are not publicly available because they contain information that could compromise research participant privacy/consent, but are available from the corresponding author on reasonable request.

## Abbreviations

CRF: Cancer-related fatigue
BLESS: Better Life after cancer, Energy, Strength, and Support Program
FACT-ES: Functional Assessment of Cancer Therapy–Endocrine Symptoms
K-PFS: Korean version of the Pittsburgh Fatigability Scale
K-PSQI: Korean version of the Pittsburgh Sleep Quality Index
K-R-PFS: Korean version of the Revised Piper Fatigue Scale
RCT: Randomized controlled trial

## References

[1] Bray F, Ferlay J, Soerjomataram I, Siegel RL, Torre LA, Jemal A (2018). Global cancer statistics 2018: GLOBOCAN estimates of incidence and mortality worldwide for 36 cancers in 185 countries. CA Cancer J Clin.

[2] Ruiz-Casado A, Álvarez-Bustos A, de Pedro CG, Méndez-Otero M, Romero-Elías M (2020). Cancer-related fatigue in breast cancer survivors: a review. Clin Breast Cancer.

[3] Schreier AM, Johnson LA, Vohra NA, Muzaffar M, Kyle B (2019). Post-treatment symptoms of pain, anxiety, sleep disturbance, and fatigue in breast cancer survivors. Pain Manag Nurs.

[4] Groenvold M, Petersen MA, Idler E, Bjorner JB, Fayers PM, Mouridsen HT (2007). Psychological distress and fatigue predicted recurrence and survival in primary breast cancer patients. Breast Cancer Res Treat.

[5] Kluger BM, Krupp LB, Enoka RM (2013). Fatigue and fatigability in neurologic illnesses: proposal for a unified taxonomy. Neurology..

[6] Eldadah BA (2010). Fatigue and fatigability in older adults. PM&R..

[7] Hoffman AJ, Brintnall RA, Given BA, Von Eye A, Jones LW, Brown JK (2017). Using perceived self-efficacy to improve fatigue and fatigability in post-surgical lung cancer patients: a pilot randomized controlled trial. Cancer Nurs.

[8] Gresham G, Dy SM, Zipunnikov V, Browner IS, Studenski SA, Simonsick EM, Ferrucci L, Schrack JA (2018). Fatigability and endurance performance in cancer survivors: analyses from the Baltimore longitudinal study of aging. Cancer..

[9] Jang MK, Kim S, Park CG, Collins EG, Quinn LT, Ferrans CE. Quality of life and prolonged symptoms in Korean breast cancer survivors. Cancer Nurs. 2020 September 25. 10.1097/ncc.0000000000000894 Online ahead of print. 10.1097/NCC.0000000000000894 33003122

[10] Hacker ED, Kim I, Park C, Peters T (2017). Real-time fatigue and free-living physical activity in hematopoietic stem cell transplantation cancer survivors and healthy controls: a preliminary examination of the temporal, dynamic relationship. Cancer Nurs.

[11] Jiang M, Ma Y, Yun B, Wang Q, Huang C, Han L (2020). Exercise for fatigue in breast cancer patients: an umbrella review of systematic reviews. Int J Nurs Sci.

[12] Zhang X, Li Y, Liu D (2019). Effects of exercise on the quality of life in breast cancer patients: a systematic review of randomized controlled trials. Support Care Cancer.

[13] Baglia ML, Lin IH, Cartmel B, Sanft T, Ligibel J, Hershman DL, Harrigan M, Ferrucci LM, Li FY, Irwin ML (2019). Endocrine-related quality of life in a randomized trial of exercise on aromatase inhibitor–induced arthralgias in breast cancer survivors. Cancer..

[14] Battaglini CL, Mills RC, Phillips BL, Lee JT, Story CE, Nascimento MG, Hackney AC (2014). Twenty-five years of research on the effects of exercise training in breast cancer survivors: a systematic review of the literature. World J Clin Oncol.

[15] Kim S, Ko YH, Song Y, Kang MJ, Lee H, Kim SH, Jeon JY, Cho YU, Yi G, Han J (2020). Pre-post analysis of a social capital-based exercise adherence intervention for breast cancer survivors with moderate fatigue: a randomized controlled trial. Support Care Cancer.

[16] Kim SH, Song YK, Han J, Ko YH, Lee H, Kang MJ, Park H, Lee H, Kim S (2020). Pro-inflammatory cytokine levels and Cancer-related fatigue in breast Cancer survivors: effects of an exercise adherence program. J Breast Cancer.

[17] Kim S, Ko YH, Song Y, Kang MJ, Lee H, Kim SH, Jeon JY, Cho YU, Yi G, Han J (2019). Development of an exercise adherence program for breast cancer survivors with cancer-related fatigue—an intervention mapping approach. Support Care Cancer.

[18] Sandel SL, Judge JO, Landry N, Faria L, Ouellette R, Majczak M (2005). Dance and movement program improves quality-of-life measures in breast cancer survivors. Cancer Nurs.

[19] Lee EH (1999). Construct validity of the revised Piper fatigue scale in Korean women with breast cancer. J Korean Acad Nurs.

[20] Cooper R, Popham M, Santanasto AJ, Hardy R, Glynn NW, Kuh D (2019). Are BMI and inflammatory markers independently associated with physical fatigability in old age?. Int J Obes.

[21] Wasson E, Rosso AL, Santanasto AJ, Rosano C, Butters MA, Rejeski WJ, Boudreau RM, Aizenstein H, Gmelin T, Glynn NW, LIFE Study Group (2019). Neural correlates of perceived physical and mental fatigability in older adults: a pilot study. Exp Gerontol.

[22] Simonsick EM, Schrack JA, Santanasto AJ, Studenski SA, Ferrucci L, Glynn NW (2018). Pittsburgh fatigability scale: one-page predictor of mobility decline in mobility-intact older adults. J Am Geriatr Soc.

[23] Oh SM, Min KJ, Park DB (1999). A study on the standardization of the hospital anxiety and depression scale for Koreans: a comparison of normal, depressed and anxious groups. J Korean Neuropsychiatr Assoc.

[24] Choi HJ, Kim SJ, Kim BJ, Kim IJ (2012). Korean versions of self-reported sleep questionnaires for research and practice on sleep disturbance. Korean J Rehabil Nurs.

[25] Fallowfield LJ, Leaity SK, Howell A, Benson S, Cella D (1999). Assessment of quality of life in women undergoing hormonal therapy for breast cancer: validation of an endocrine symptom subscale for the FACT-B. Breast Cancer Res Treat.

[26] Juvet LK, Thune I, Elvsaas IØ, Fors EA, Lundgren S, Bertheussen G, Leivseth G, Oldervoll LM (2017). The effect of exercise on fatigue and physical functioning in breast cancer patients during and after treatment and at 6 months follow-up: a meta-analysis. Breast.

[27] Abrahams HJ, Gielissen MF, Verhagen CA, Knoop H (2018). The relationship of fatigue in breast cancer survivors with quality of life and factors to address in psychological interventions: a systematic review. Clin Psychol Rev.

[28] Xiao C, Miller AH, Felger J, Mister D, Liu T, Torres MA (2017). Depressive symptoms and inflammation are independent risk factors of fatigue in breast cancer survivors. Psychol Med.

[29] Tchen N, Juffs HG, Downie FP, Yi QL, Hu H, Chemerynsky I, Clemons M, Crump M, Goss PE, Warr D, Tweedale ME (2003). Cognitive function, fatigue, and menopausal symptoms in women receiving adjuvant chemotherapy for breast cancer. J Clin Oncol.

